# Supplementary material for: Density estimates of monarch butterflies overwintering in central Mexico
Source: PeerJ. 2017 Apr 26;5:e3221. doi: 10.7717/peerj.3221 (PMC5408724; doi:10.7717/peerj.3221)
Supplement: Appendix S1 [file peerj-05-3221-s001.docx]

Appendix A. Density estimates of monarch butterflies overwintering in central Mexico: Modeling of Brower et al. (2004) storm-mortality data.

Data provided by L. Brower.


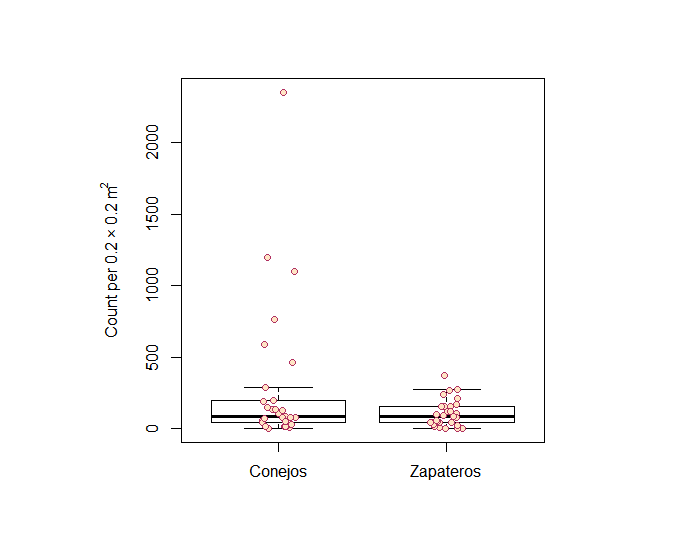


Counts of dead and moribund butterflies made at Conejos colony.

Min. 1st Qu. Median Mean 3rd Qu. Max.

1.0 43.0 86.0 290.4 195.0 2354.0

Zapatero colony

Min. 1st Qu. Median Mean 3rd Qu. Max.

0.0 40.0 83.0 105.1 158.0 372.0

Three of the 29 samples collected for the Conejos colony comprised 55% of the total number of individuals collected; conversely, the three highest counts for the Zapatero colony comprised 28% of the total number of individuals collected. The maximum Conejos colony observation (2,354 butterflies per 0.2 × 0.2 m^2^ plot) was identified as an outlier among the 29 samples; Grubbs test identified the 2^nd^ highest Conejos observation, 1,100, as an outlier as well.

> chisq.out.test(conejosmd, variance=var(conejosmd), opposite = FALSE) #checking to see if highest value is an outlier

chi-squared test for outlier

data: conejosmd

X-squared = 16.655, p-value = 0.00004483

alternative hypothesis: highest value 2354 is an outlier

> dixon.test(conejosmd, type = 0, opposite = FALSE, two.sided = FALSE)

Dixon test for outliers

data: conejosmd

Q = 0.53672, p-value < 0.00000000000000022

alternative hypothesis: highest value 2354 is an outlier

> grubbs.test(conejosmd, type = 20, opposite = FALSE, two.sided = FALSE)

Grubbs test for two outliers

data: conejosmd

U = 0.24417, p-value < 0.00000000000000022

alternative hypothesis: highest values 1199 , 2354 are outliers

Similarly, 372 and 275 butterflies collected in single 0.2 × 0.2 m^2^ plots were identified as outliers in the Zapatero sample.

Because of these outliers, or extreme values, we used the *evd* library in R (Stephenson, A. G. 2002. evd: Extreme Value Distributions. R News 2(2):31-32, URL: <http://CRAN.R-project.org/doc/Rnews/>) for fitting theoretical distributions with maximum likelihood to the generalized extreme value distribution. A gamma distribution fit best

> fitzap_g$aic

[1] 307.1915

> fitzap_ln$aic

[1] 344.2689

> fitzap_w$aic

[1] 316.2081

The location of this gamma distribution for the Zapatero colony is 54.132, the scale 56.841, and the shape 0.299.

fitzapev <- fgev(zapateromd)

param2z <- fitzapev$estimate

> param2z

loc scale shape

54.1324358 56.8411528 0.2986697

Zapateros


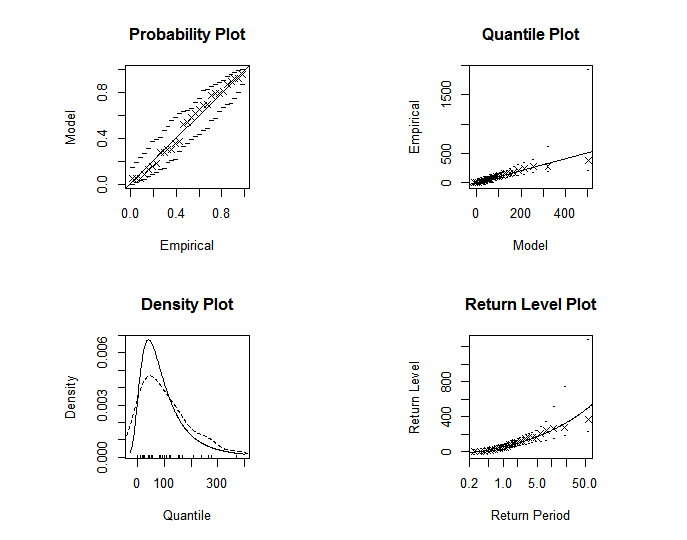


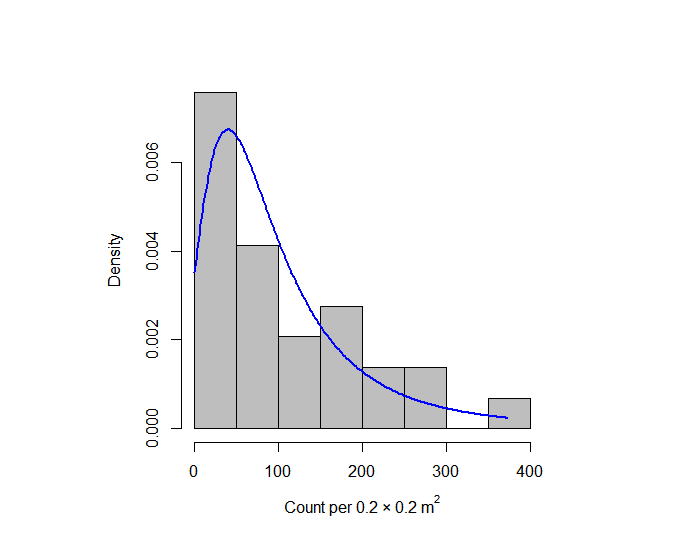


For the Conejos colony, a lognormal model fit the distribution best (AIC = 377.984 versus AIC = 379.741 for a Weibull fit).

fitcon_lnr <- fitdistr(conejosmd, "lognormal")

> fitcon_lnr

meanlog sdlog

4.5395633 1.6315071

(0.3029633) (0.2142274)

Conejos


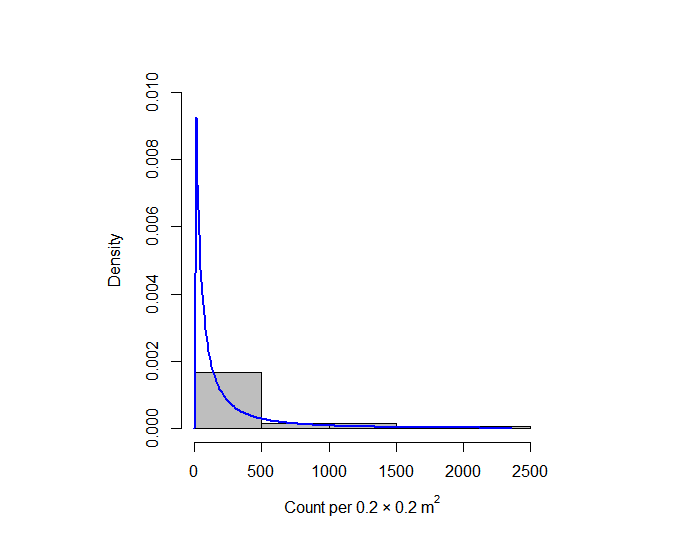


Conejos


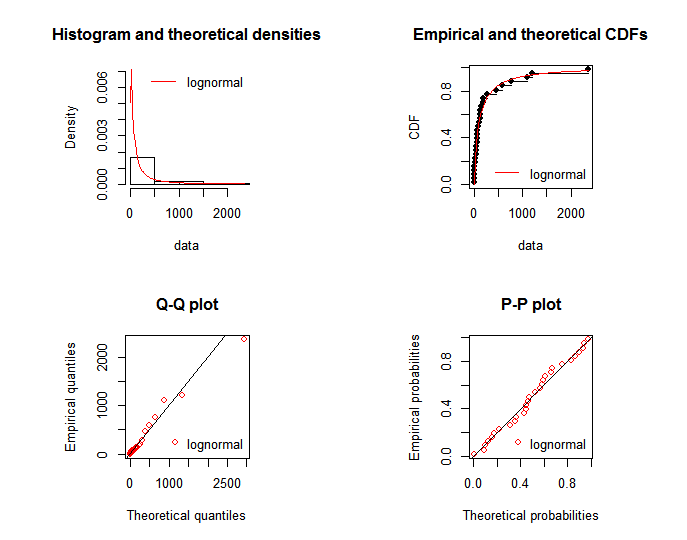


Simulating from the Zapatero colony generalized extreme value distribution yielded

> summary(thetaZapateroEV)

Min. 1st Qu. Median Mean 3rd Qu. Max.

0.00 36.51 76.14 110.90 140.00 4000.00

This compares to an observed median of 83 butterflies and an observed mean of 105.1 butterflies (variance 8,996) from the 29 samples collected from Zapatero colony.

There are 250,000 0.2 × 0.2 m^2^ plots per hectare. Multiplying values drawn from the distribution by 250,000 provides the estimates in densities per ha.

> summary(thetaZapateroEVHA)

Min. 1st Qu. Median Mean 3rd Qu. Max.

0 9110000 19000000 27700000 35000000 3210000000

Note, the maximum is 3.2 billion per ha according to this extreme value distribution, which is absurd (and further evidence that the maximum observations were outliers). The median, however, is 19 million per ha. The mean is 27.7 million per ha. Placing a maximum density of 100,000,000 per ha led to a slightly lowered mean.

> summary(thetaZapateroEVHA)

Min. 1st Qu. Median Mean 3rd Qu. Max.

0 8750000 18200000 23500000 32700000 100000000

The revised median is 18.2 million butterflies per ha and the mean is 23.5 million butterflies per ha.

Simulating from the Conejos colony log-normal distribution yielded

> summary(thetaConejos)

Min. 1st Qu. Median Mean 3rd Qu. Max.

0.0 31.1 93.7 353.9 281.5 390600.0

These values compare to a median of 86 butterflies per sample and a mean of 290.4 butterflies per sample (variance = 255,674).

When extrapolating from the plots to the hectare scale, the distribution (capped with values no greater than 100,000,000) is

> summary(thetaConejosHA)

Min. 1st Qu. Median Mean 3rd Qu. Max.

12500 6060000 15900000 25000000 37000000 100000000

Thus, the median density for this colony was 15.9 million butterflies per ha, with a mean of 25 million butterflies per ha.
