# Supplementary material for: Density estimates of monarch butterflies overwintering in central Mexico
Source: PeerJ. 2017 Apr 26;5:e3221. doi: 10.7717/peerj.3221 (PMC5408724; doi:10.7717/peerj.3221)

Appendix B. Density estimates of monarch butterflies overwintering in central Mexico: lognormal distribution resulting from the mixture of six data sources.


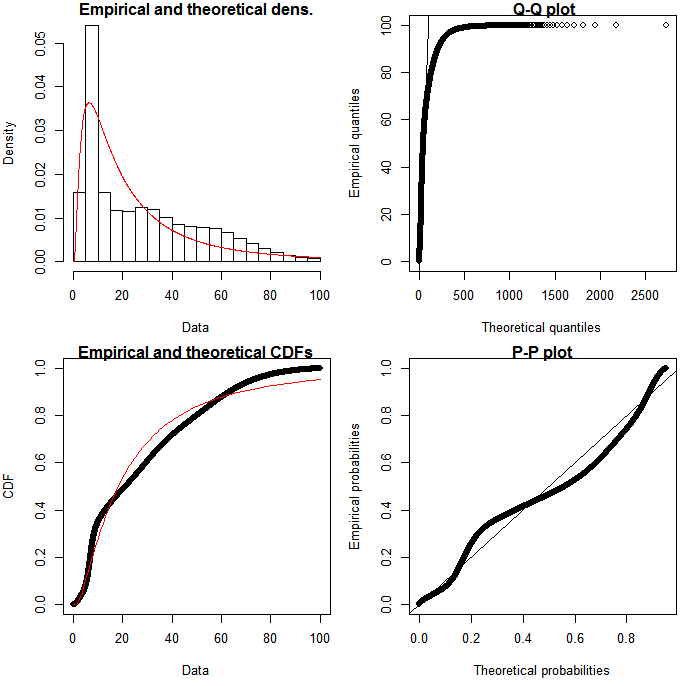

Supplement: Appendix S2 [file peerj-05-3221-s002.docx]
